# Characterizing the nectar microbiome of the non-native tropical milkweed, *Asclepias curassavica*, in an urban environment

**DOI:** 10.1371/journal.pone.0237561

**Published:** 2020-09-02

**Authors:** Magdalena L. Warren, Karin E. Kram, Kathryn E. Theiss

**Affiliations:** 1 Department of Biology, California State University Dominguez Hills, Carson, California, United States of America; 2 Department of Biology, Stanford University, Stanford, California, United States of America; Instituto Nacional de Pesquisas da Amazonia, BRAZIL

## Abstract

In increasingly urban landscapes, the loss of native pollen and nectar floral resources is impacting ecologically important pollinators. Increased urbanization has also brought about the rise of urban gardens which introduce new floral resources that may help replace those the pollinators have lost. Recently, studies have shown that the microbial communities of nectar may play an important role in plant-pollinator interactions, but these microbial communities and the floral visitors in urban environments are poorly studied. In this study we characterized the floral visitors and nectar microbial communities of *Ascelpias curassavica*, a non-native tropical milkweed commonly, in an urban environment. We found that the majority of the floral visitors to *A*. *curassavica* were honey bees followed closely by monarch butterflies. We also found that there were several unique visitors to each site, such as ants, wasps, solitary bees, several species of butterflies and moths, Anna’s hummingbird, and the tarantula hawk wasp. Significant differences in the nectar bacterial alpha and beta diversity were found across the urban sites, although we found no significant differences among the fungal communities. We found that the differences in the bacterial communities were more likely due to the environment and floral visitors rather than physiological differences in the plants growing at the gardens. Greater understanding of the impact of urbanization on the nectar microbiome of urban floral resources and consequently their effect on plant-pollinator relationships will help to predict how these relationships will change with urbanization, and how negative impacts can be mitigated through better management of the floral composition in urban gardens.

## Introduction

The breadth and persistence of native pollinators is vital to the future of agricultural productivity, biodiversity, and ecosystem services in North America [1–4]. The processes behind the declines in native pollinators are numerous and include global climate change [5] and habitat conversion [6]. Within the United States, the urban environment is the fastest growing land use category [7], and may act as refugia for pollinators [8,9]. As the landscape becomes more urbanized, the built environment has the potential to become an important resource for insect pollinators. Most insect pollinators rely on the nectar provided by flowers as their primary sources of nutrition [10,11]. Floral and pollinator diversity across the built environment varies at multiple scales, from localized backyards and urban gardens to broad scales across the full urbanization gradient [12,13]. Urban plantings can actually increase pollinator resources because the plants often have a longer flowering time due to increased resource availability as compared to natural areas [14]. Additionally, the urban environment is generally warmer than the surrounding natural areas, altering the phenology of native plant species, including increasing floral density, when compared to the local natural areas [15–19]. Insect pollinators, including bees, may heavily rely on these resources when there is high resource variability in the surrounding environments [20]. These factors make the urban environment a vital resource for pollinators.

The floral diversity within the urban environment typically contains both native and non-native species [21], yet the full consequences of this mix have not been thoroughly studied. Increased native plant species within the built environment may benefit insect pollinators, especially bees [22], while non-native species may extend the availability of important pollinator resources such as nectar on a temporal basis, altering pollinator behavior [14]. Nectar sources in urban gardens, different from those of the original native flora, might introduce a new community of microorganisms that may interact differently with the existing plants and pollinators in the region. Since the plants in urban gardens have an increasing contribution to the sustaining the diversity of animal pollinators [8,15,23], understanding their microbiome will be of utmost importance.

Floral nectar is home to a diverse group of microorganisms, including fungi and bacteria [24–28]. These microorganisms are introduced into nectar through floral visitors [29–31], and the type of floral visitor may influence the microbes introduced into the nectar [30], potentially feeding back to affect plants and pollinators through microbial modification of nectar chemistry. Once microorganisms colonize nectar, they may compete for amino acids and facilitate or inhibit the colonization of other microbes [25,32]. As microbial communities develop, they change the nectar’s properties in several ways, such as decreasing hydrogen peroxide concentration [33], decreasing pH, changing the concentration and composition of sugars and amino acids [33–35], or increasing the floral temperature [24]. These changes can consequently affect plant-pollinator relationships [33,36–39]. For example, nectar colonized by yeast may attract pollinators [37,40] or may not have an effect on pollinator attraction, whereas bacterial colonizers commonly decrease nectar attractiveness [33,38] likely through changes in the chemical composition of the nectar [39]. To our knowledge, however, most of these studies have been conducted with plants located in natural environments [26,30,31,41].

A common flowering plant found in urban gardens, *Asclepias curassavica* L. (Apocynaceae), is an ideal candidate for nectar microbiome studies in urban areas. Due to its aesthetic appeal and service as a food resource for the monarch butterfly, *Danaus plexippus* L. [42,43], *A*. *curassavica* is often found in the butterfly gardens in urban landscapes where it can serve as a resource not only for *D*. *plexippus* but potentially for a variety of animal-pollinators [14,44–46]. Using *A*. *curassavica* in urban gardens throughout the Los Angeles, California urban center, we aimed to characterize its interactions with floral visitors and nectar microbes. First, we focused on describing who the floral visitors of *A*. *curassavica* were and how they differed across the urban environment of Los Angeles. Second, we identified the fungal and bacterial communities found in the nectar across this environment to describe similarities and differences across our sites. Third, we explored how differences in microbial communities were correlated with differences in *A*. *curassavica* plants grown in different environments.

## Materials and methods

### Study sites

We selected three highly diverse urban botanical gardens where *A*. *curassavica* was flowering along a west-east transect of the Los Angeles metropolitan area: the South Coast Botanic Garden (SCBG) located on the coast of California (33° 78´ N, 118° 35´ W), the California State University, Fullerton Arboretum (CSUF), found in the center of the urban core (33° 89´ N, 117° 32´ W), and the University of California, Riverside, Botanic Gardens (UCR) located at the eastern edge of the urban area (33° 97´ N, 117° 32´ W). We visited SCBG on June 4, 2017, a cloudy day with intermittent breaks of sunshine, where the temperature fluctuated from 17.2 to 20°C. On June 8, 2017 we visited CSUF where the temperature ranged from 22.78 to 33.94°C on a very sunny day. Lastly, we visited UCR on June 11, 2017, a sunny day when temperatures ranged from 22.5 to 31.17°C. Pollinator observations were conducted for approximately seven hours each day, and floral and nectar sampling was conducted as follows.

### Plant selection

**Fig 1:**
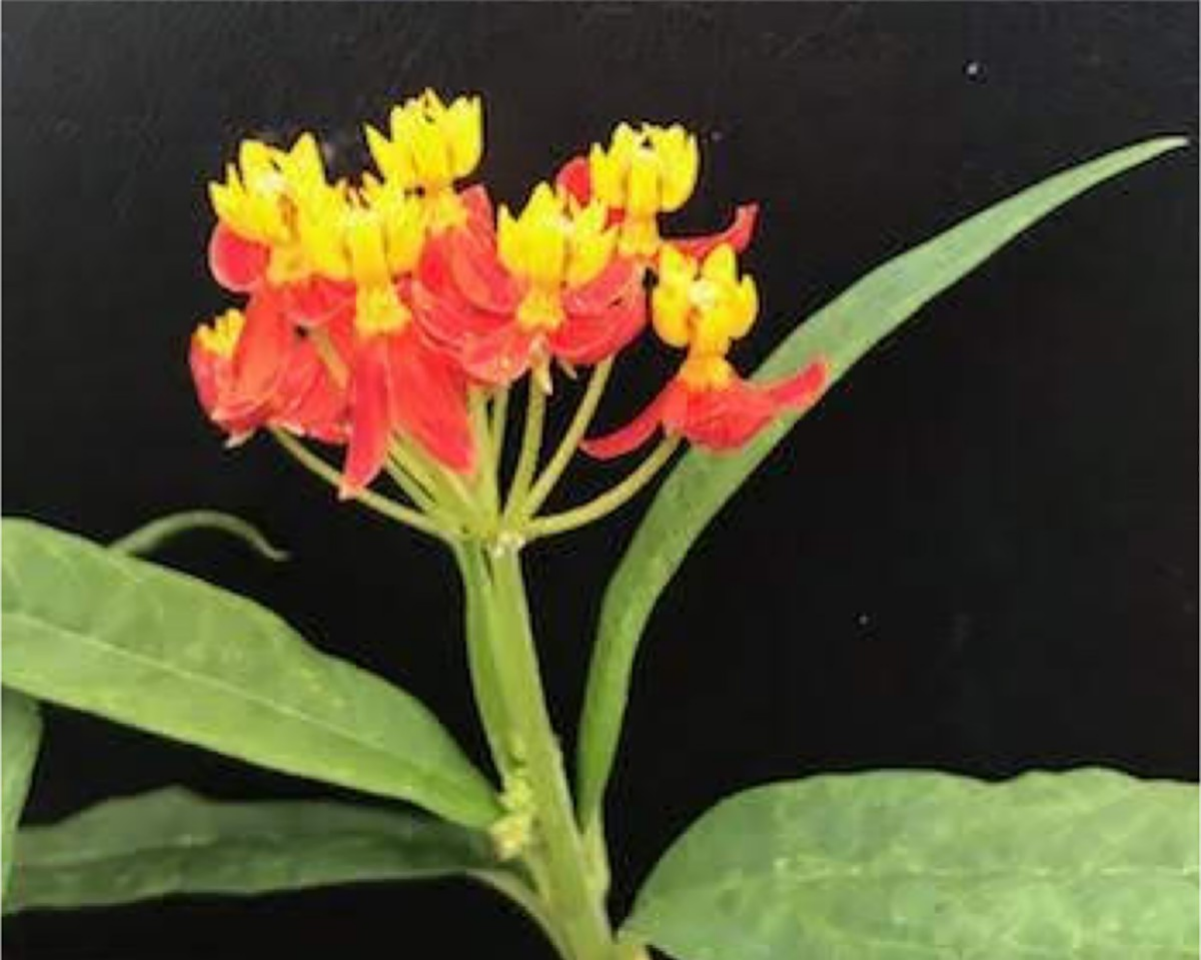
*Asclepias curassavica* umbel with 7 flowers. Photo credit: Kathryn E. Theiss.

*Asclepias curassavica* is a perennial herb, typically blooming from June until October, and its umbels are organized into umbels of 6–15 flowers per umbel [47]. Each flower has five sepals colored bright red or orange and five modified petals that are typically bright yellow in coloring. These petals form cup-like shapes known as cuculli that fill up with nectar [48] (Fig 1), with each flower producing 1–2μl of nectar per day [49]. The nectar of *A*. *curassavica* consists of high amounts of glucose, sucrose, and fructose sugars [49], amino acids, and potentially cardenolides [47] although the concentration of cardenolides has been found to be in low concentration or potentially non-existent in the nectar [50]. Insects taking advantage of *A*. *curassavica* nectar insert their proboscises or tongues into the cup-like petals, extracting the nectar while the plant’s pollinia stick to their legs, ready to be transported to another plant [48], sealing the mutualistic relationship between *A*. *curassavica* and its pollinators. These floral visitors have the potential to introduce various microbes into the nectar of *A*. *curassavica* by not only inserting their mouth parts, but also their feet and legs into the nectar.

To ensure that we had plenty of newly opened flowers, we used a combination of commercially propagated plants and plants that were already established at each sampling location. Twenty-four *A*. *curassavica* plants were purchased from commercial growers and grown in the greenhouse at California State University Dominguez Hills (CSUDH). We placed the plants in a mesh tent to prevent floral visitors from coming into contact with new flowers in order to control for nectar sterility. Three days before the pollinator observations at each of the three sites, the established *A*. *curassavica* plants were inspected for umbels with large buds. We covered these umbels with organza mesh bags to keep insects from interacting with the flowers as they opened. Any flowers that were already open in these umbels were removed to eliminate any samples with unknown floral visitors. On the day of pollinator observations at each site, we selected five to six plants from the CSUDH population with open umbels of eight or more flowers and transported them to the study site to increase the total available umbels. The plants from the greenhouse were distributed randomly between the plants growing at the site. Each of the 32 target umbels was labeled with neon yellow tape for identification.

### Floral visitor observations

At the beginning of the observation period, temperature and cloud cover were recorded, and these measurements were repeated every 30 minutes throughout the entire observation period. We, the observers, were situated at least one meter from the target umbels so as to not affect floral visitor behavior. We recorded the time at which a floral visitor came in contact with the nectar of one of the flowers of a tagged umbel. Each contact with an umbel was treated as a new visit, even if it was the same visitor that had left the original umbel, gone to another, and then returned. The observations ended between 4pm and 5pm depending on the hours of operation of the site. At the end of the observation period, the tagged umbels were once again covered with organza bags and the plants were left at the site to re-accumulate nectar overnight.

### Nectar extraction

The morning following the floral visitor observation, we extracted the nectar using a sterile allergy syringe (Becton Dickinson and Company, Franklin Lakes, New Jersey, CA, USA). At CSUF and UCR, we collected the nectar on site and stored it on ice for transport back to the lab. Since SCBG is located close to the lab, the target umbels from SCBG were removed from the plants, and transported back to the lab before nectar was extracted. Due to the low yield (1–2 μl of nectar per flower; 49) and uncertainty of floral visitor contact with specific flowers, nectar from all flowers in one umbel was combined in a single 1.7 mL microcentrifuge tube.

### Bacterial and fungal DNA sequencing

For each of the 32 samples per site, 1μL of nectar from the original nectar sample was combined with 9μL of Milli-Q sterile water in a 1.7mL microcentrifuge tube for DNA extraction. DNA was extracted from all the samples using the Qiagen DNeasy Blood and Tissue kit following the Gram-positive bacteria protocol (Qiagen, Germantown, Maryland, USA). On average, we extracted 1.41 ng/μl of DNA from each nectar sample. The V4 region of the bacterial 16S ribosomal RNA gene and the fungal internal transcribed spacer 1 region (ITS1) were amplified with Illumina adapters on the 5’ ends of both forward and reverse primers (Table 1). Each 25μL PCR reaction consisted of 1x (12.5μL) KAPA HiFi HotStart ReadyMix (Kapa Biosystems, Wilmington, Massachusetts, USA), 1μM of each primer, and 10.5μL of extracted DNA. For each nectar sample, bacterial and fungal DNA regions were amplified separately. First round PCR conditions for the amplification of the 16S V4 region were 3 minutes at 95°C, followed by 25 cycles of 30 seconds at 95°C, 30 seconds at 55°C, and 30 seconds at 72°C, and finalized with 5 minutes at 72°C. The ITS1 gene region first round PCR conditions were 1 minute at 94°C, followed by 30 cycles of 30 seconds at 94°C, 30 seconds at 52°C, and 30 seconds at 72°C, with a final step of 7 minutes at 72°C. The resulting amplicons were purified with AMPure XP beads (Agencourt, Beverly, Massachusetts, USA), and then a second round of PCR was conducted to concatenate the 8bp index (S1 Table) sequences and the Illumina sequencing adaptors. The second round PCR conditions were identical for both gene regions: 3 minutes at 95°C, followed by 8 cycles of 30 seconds at 95°C, 30 seconds at 55°C, and 30 seconds at 72°C, and finished with 5 minutes at 72°C. DNA concentration and quality of the pooled amplicon libraries were analyzed using a Fragment Analyzer (Agilent, Santa Clara, CA, SA), and then sequenced using an Illumina V2 2 x 300 bp paired-end sequencing kit on an Illumina MiSeq sequencer at CSUDH with a 15% PhiX spike-in [51].

**Table 1:** Adaptors and primers used for first round PCR.

	Forward	Reverse
**16S rRNA (V4)**	515f (5’-GTGYCAGCMGCCGCGGTAA-3’) [52]	806r (5’-GGACTACNVGGGTWTCTAAT-3’) [52]
**ITS1**	ITS1f (5’-CTTGGTCATTTAGAGGAAGTAA -3’) [53]	ITS2 (5’-GCTGCGTTC TTCATCGATGC-3’) [53]
**Illumina adaptor**	5’-TCGTCGGCAGCGTCAGATGTGTATAAGAGACAG-3’	5’-GTCTCGTGGGCTCGGAGATGTGTATAAGAGACAG-3’

Forward and reverse sequences of 16S rRNA primers used to amplify bacterial species and ITS1 primers used to amplify fungal species. The Illuminar adaptors were applied to both PCR products.

The raw data from the Illumina MiSeq sequencing run was processed using the Claident pipeline [54]. To ensure samples were identified properly, the raw MiSeq BCL data was converted into FASTQ data with the Illumina bcl2fastq v1.8.4 program (Illumina, San Diego, California, USA) and then demultiplexed within Claident [55]. Resulting sequencing reads with low quality scores of less than 30 were deleted. The program PEAR v0.9.6 [56] was used to merge the forward and reverse reads of each sample together. The merged reads were then filtered for quality, and merged reads with a quality score of less than 30 or length of less than 150 base pairs were deleted. UCHIME v4.2 [57] was used to remove chimeric reads from the data, and the resulting reads that had passed all the aforementioned filtering steps were clustered into operational taxonomic units (OTUs) using the program VSEARCH [58], with a minimum sequence similarity threshold of 97 percent. Bacterial and fungal taxonomy was assigned to the OTUs using the RDP Naïve Bayesian rRNA Classifier v2.11 which was either trained on the 16S rRNA training set 16 [59] for bacterial identification or the Warcup Fungal ITS trainset 2 [60] for fungal identification. For each dataset, any taxonomic assignments not in the kingdom bacteria were eliminated from the bacterial dataset, and any taxonomic assignments not in the domain fungi were eliminated from the fungal dataset. The bacteria OTU table was rarefied to 2000 sequencing reads before any further analysis was done [61]. Due to the low number of fungal sequencing reads, the resulting OTU table was rarefied to 100. After rarefaction, only 63 fungal samples contained at least 100 reads and persisted after rarefaction, in contrast to the 90 of 96 bacterial samples that remained after rarefying the bacterial OTU table.

### Data analysis

The bacterial and fungal communities were analyzed separately. All analyses were done in *R* version 3.5.0 [62]. The differences in class level alpha diversity using observed richness, Chao1, and Shannon diversity indices were visualized (with R package *phyloseq* [61]). To assess the significance of the differences in the bacterial and fungal alpha diversities we fit a generalized linear mixed model (GLMM) based on a negative binomial distribution with the plant identity as a random effect (with R package *glmmTMB* [63]) to the relationship between observed class richness and the site from which the sample was collected. To control for any impacts of the individual plant used, we added plant identification as a random effect to account for any pseudo-replication in the samples. To explore which microbial taxa might be influencing the differences in alpha diversity the relative abundance of microbial taxa was analyzed by first aggregating the microbial communities to the class level, and then modeling the abundance of each genus as a function of site. An analysis of variance and Tukey honest significant differences *post hoc* tests (with R package *stats* [62]) were done on the linear model to explore interesting trends. To analyze the beta diversity differences between the samples at each site, the microbial communities were aggregated to the class taxonomic level, ordinated based on Bray-Curtis dissimilarity matrices (with R packages *vegan* [64] and *phyloseq* [61]) and were visualized using a non-metric multidimensional scaling (NMDS). The effects of site on the nectar microbial class community composition was tested using a permutational multivariate analysis of variance (PERMANOVA with 999 permutations) [65], and the significance of canonical axes of the Bray-Curtis dissimilarity matrices were analyzed using a permutation test for redundancy analysis [66].

## Results

### Floral visitors

In total, the umbels at SCBG were visited 442 times, those at CSUF 711 times, and those at UCR 689 times. The ratio of the number of floral visitors to the number of flowers in a given umbel from which the nectar sample was derived was significantly different only between SCBG and CSUF (two sample t-test, P < 0.05), but not significantly different between UCR and either SCBG (two sample t-test, P = 0.380) or CSUF (two sample t-test, P = 0.053). The majority of the floral visitors to all three sites were honey bees, *Apis mellifera* L., making up 84.6%, 87.2%, and 96.2% of the floral visits at SCBG, CSUF, and UCR respectively. The other two most abundant visitors at the three sites were monarchs, *D*. *plexippus*, and flies (Muscidae) that we were unable to identify to species. Of the 96 umbels we observed, 29 had only honey bees as visitors, 66 had a mix of honey bees and other visitors, and only 1 umbel had no honey bee visitors. Although the classification of visitors to the umbels at all three sites were similar, some were unique to each site. Of the three sites, only SCBG umbels were visited by beetles (*Diabrotica* sp.), wasps (*Polistes* sp.), and solitary bees (Apidae). Unique to CSUF was the Western tiger swallowtail (*Papilio rutulus* L.), and to UCR were the funereal duskwing (*Erynnis funeralis* Scud. & Burg.), the marine blue butterfly (*Leptotes marina* Reakirt), Anna’s hummingbird (*Calypte anna* Lesson), and the tarantula hawk wasp (*Pepsis grossa* Fabricius). Overall, 11 different types of insects visited the umbels at SCBG, six different types visited those at CSUF, and eight different types visited those at UCR (S2 Table).

### Alpha diversity

**Fig 2:**
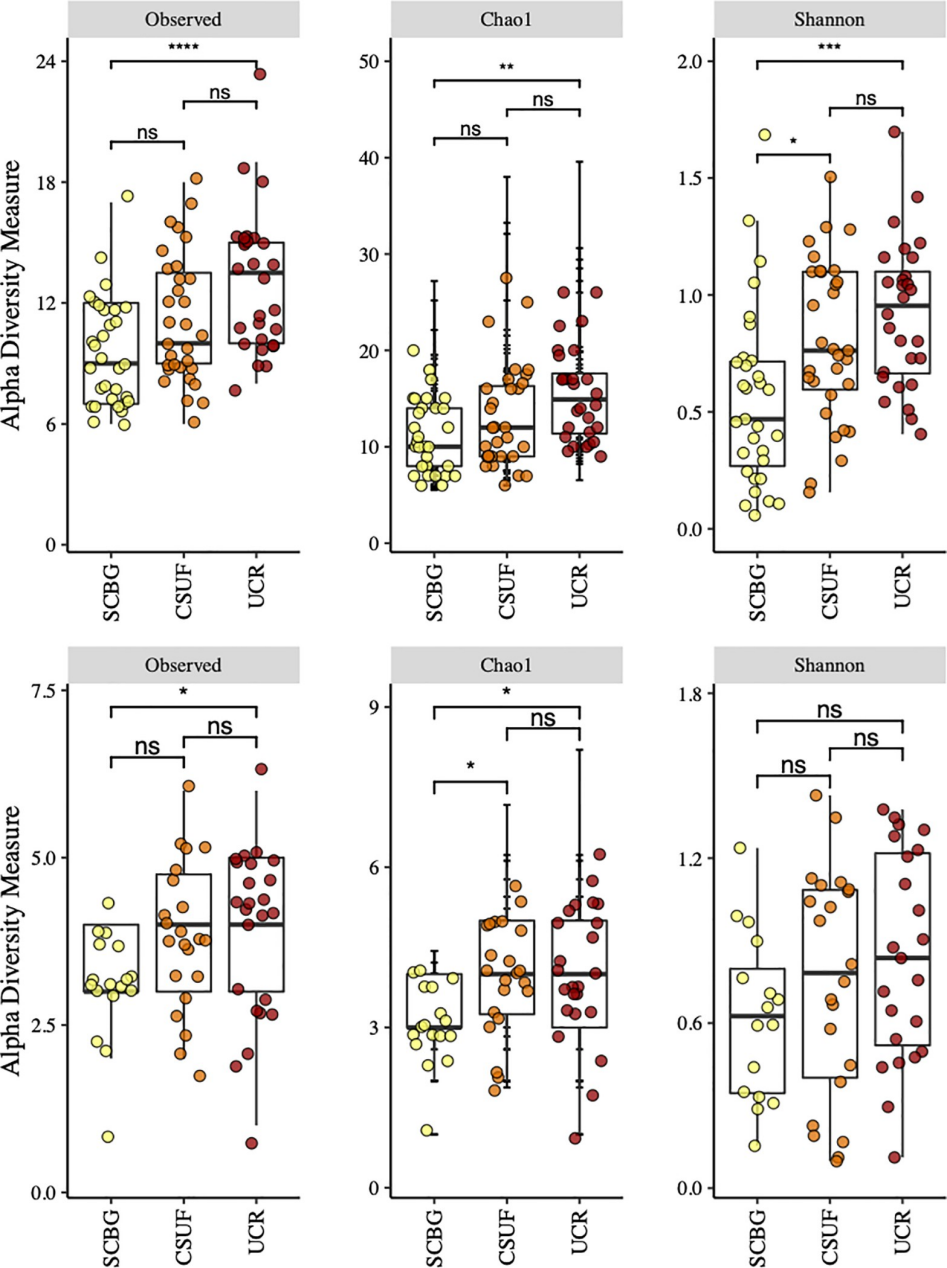
Bacterial and fungal alpha diversity from SCBG, CSUF, and UCR nectar samples. Bacterial alpha diversity was measured using (A) the observed class richness, (B) the Chao1 index, and (C) the Shannon diversity index. Fungal alpha diversity was similarly measured with (D) the observed class richness, (E) the Chao1 index, and (F) the Shannon diversity index.

The bacterial alpha diversity gradually increases from SCBG to UCR in all three measurements (Fig 2A–2C). We found that the alpha diversity at CSUF was greater than that at SCBG (*P* < 0.01). However, UCR exhibited the highest alpha diversity of the three sites, significantly more than that at SCBG (*P* < 0.001). In contrast, the species richness at CSUF and UCR were fairly similar (*P* = 0.420). In the fungi, there was no significant difference in class richness among the three sites (Fig 2D–2F).

We further examined the bacterial and fungal OTUs that were more than 1% abundant across all samples. Of special interest were the bacterial OTUs identified to the genera *Acinetobacter* and *Neokomagataea* (formerly *Gluconobacter*), both known nectar specialists [67], and the fungal OTUs identified to *Aureobasidium*. *Acinetobacter* species were present in 95 of the 96 samples, *Neokomagataea* species were present in 57 of the 96 samples, and *Aureobasidium* species were present in 70 of the 96 samples.

**Fig 3:**
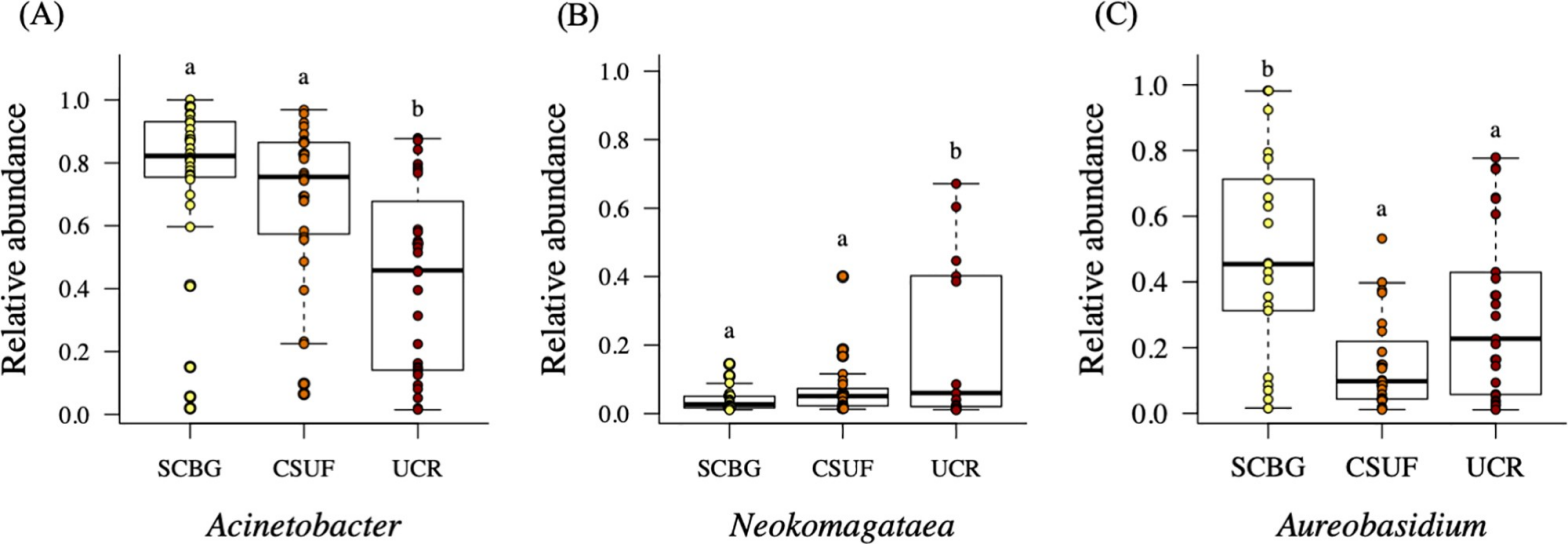
Relative abundance (number of reads per sample/ total number of reads per sample) of targeted microbes at SCBG, CSUF, and UCR. (A) *Acinetobacter*; (B) *Neokomagataea*; (C) *Aureobasidium*. Letters above bar indicate abundances that differ significantly (Tukey HSD test, *P <* 0.05).

*Acinetobacter*, for which about 73% of the sequences were identified as *A*. *nectaris* and about 25% identified as *A*. *boissieri*, was significantly lower in its relative abundance in the bacterial communities from UCR when compared to the other two sites (Tukey HSD, UCR to SCBG *P* < 0.001, UCR to CSUF *P* < 0.01; Fig 3A). The other genus, *Neokomagataea*, showed contrasting trends to those observed in the *Acinetobacter* relative abundances. Like *Acinetobacter*, we found that the *Neokomagataea* displayed a similar relative abundance at SCBG and CSUF (Tukey HSD P = 0.876; Fig 3B), however they were much less abundant than *Acinetobacter* at both sites. At UCR, where the relative abundance of *Acinetobacter* dropped, the relative abundance of *Neokomagataea* increased significantly (Tukey HSD, UCR to SCBG *P* < 0.01, UCR to CSUF *P* < 0.001; Fig 3B). On average, samples with *Neokomagataea* present at UCR had more than 40% of the community composed of this single genus. Where *Acinetobacter* was found as a member of the community, at any of the three sites, it dominated the community making up, on average, at least 50% of the community, whereas *Neokomagataea* only dominated in the UCR nectar bacterial communities. Also, the number of samples containing *Neokomagataea* decreased by almost half from SCBG (21 out of 32 samples) and CSUF (23 out of 32 samples) to UCR, where only 13 out of 32 samples contained *Neokomagataea*. *Aureobasidium*, the only abundant fungus that showed differences among the three sites, decreased significantly in relative abundance between SCBG and CSUF (Tukey HSD *P* < 0.001) and SCBG and UCR (Tukey HSD *P* < 0.05), but was not significantly different between CSUF and UCR (Tukey HSD *P* = 0.086; Fig 3C).

**Fig 4:**
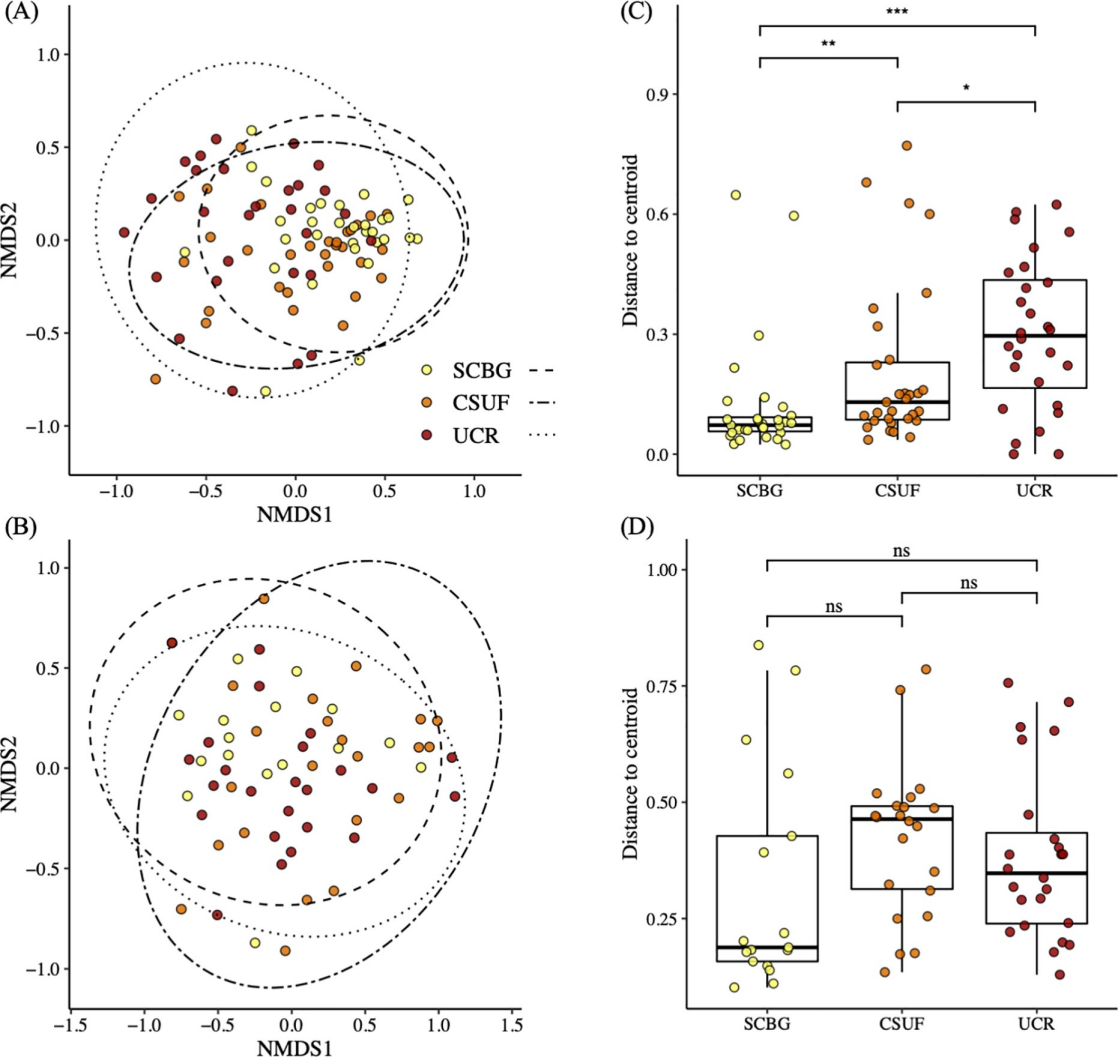
NMDS ordination of bacterial and fungal community compositions and the effect of site location on the β diversity in the microbial communities. All data was aggregated to the class taxonomic level for this analysis and each point represents the bacterial community in one nectar sample. (A) The NMDS ordination of the bacterial communities and (B) the NMDS ordination of the fungal communities have ellipses that show the 95% confidence interval. (C) The distance to centroid of each bacterial community and (D) the distance to centroid of each fungal community were calculated in multivariate space using betadisper. “***” *P <* 0.001, “**” *P <* 0.01, “*” *P <* 0.05 based on Wilcoxon signed-ranks test for pairwise comparisons.

We found that the ordination of the bacterial communities, aggregated to the class taxonomic level, exhibited significant differences in the bacterial compositions of these three sites (PERMANOVA F_2,87_ = 10.943, *P* < 0.001, R^2^ = 0.201; Fig 4A). Using the Wilcoxon signed-rank test, we found that the analysis of the distance to centroid of the ordination showed a significantly greater variance in the composition of the bacterial communities from UCR compared to the other two sites, and SCBG had significantly smaller variance in composition than both CSUF and UCR (SCBG to CSUF *P* < 0.01, CSUF to UCR *P* < 0.05, SCBG to UCR *P* < 0.001; Fig 4C). In contrast, in the fungal community ordination plot, once again aggregated to the class taxonomic level, all the samples seemed to cluster close to each other, regardless of the location from which the sample was derived with little difference in fungal composition among the three sites (PERMANOVA F_2,60_ = 2.944, *P* = 0.019, R^2^ = 0.089; Fig 4B). Also, the variance for each of the sites was similar, with no obvious clustering of points from any of the three locations. This is clear in the analysis of the distance to the centroid of the multi-dimensional points, where the Wilcoxon signed-rank test showed no significant difference between the three sites (SCBG to CSUF *P* = 0.063, SCBG to UCR *P* = 0.088, CSUF to UCR *P* = 0.242; Fig 4D).

**Fig 5:**
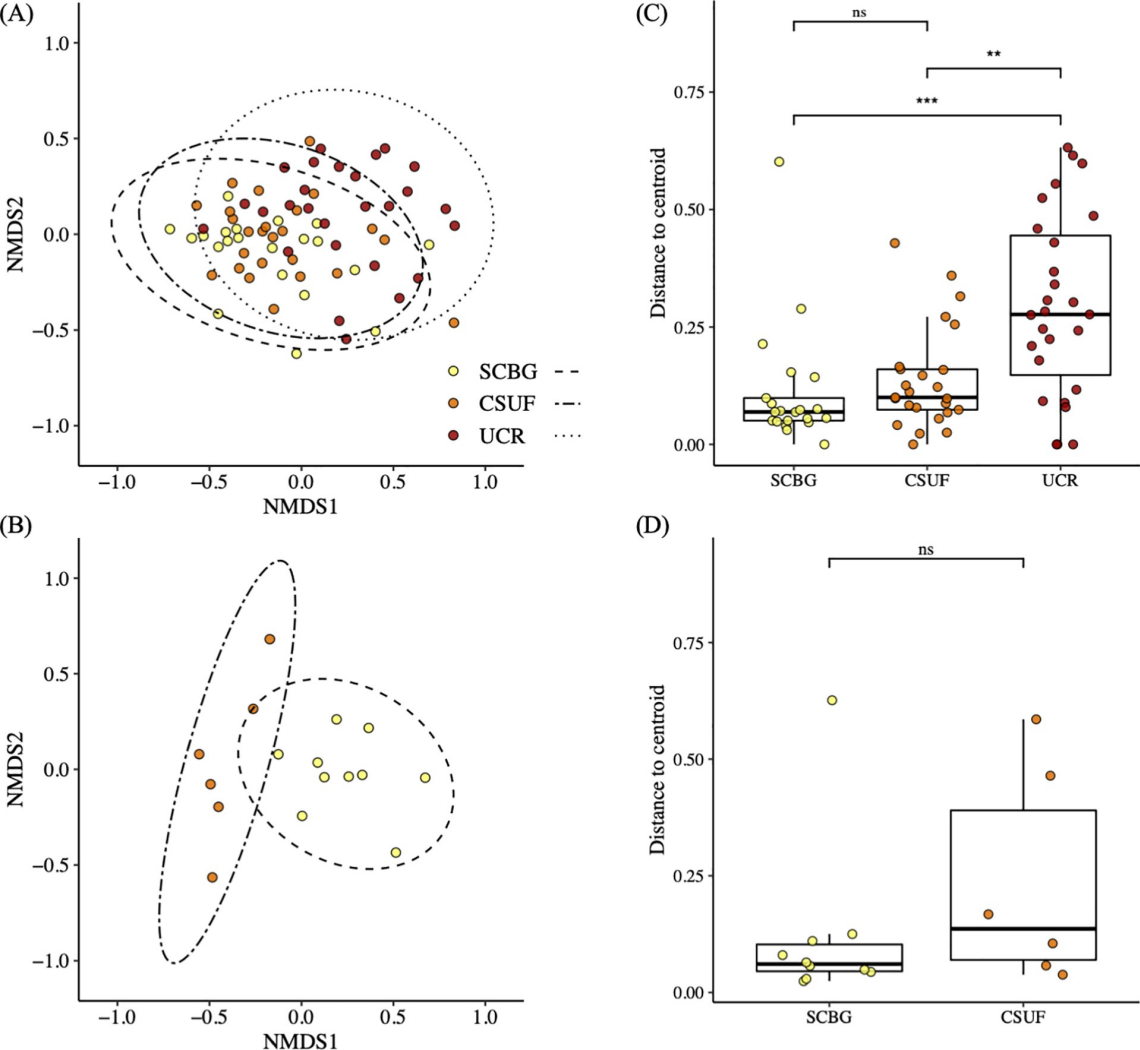
NMDS ordination of bacterial and fungal community compositions and the effect of site location on the β diversity in the microbial communities. All data was aggregated to the class taxonomic level for this analysis and each point represents the bacterial community in one nectar sample. (A) The NMDS ordination of the composition of the bacterial communities from nectar extracted from plants grown at CSUDH and (B) the NMDS ordination of the composition of the bacterial communities of samples taken from plants grown at both the SCBG and CSUF sites have ellipses that show the 95% confidence interval. (C) The distance to centroid of each bacterial community from CSUDH grown plants and the (D) distance to centroid of each bacterial community from the SCBG and CSUF grown plants were calculated in multivariate space using betadisper. “***” *P <* 0.001, “**” *P <* 0.01, “*” *P <* 0.05 based on Wilcoxon signed-ranks test for pairwise comparisons.

At each site, more than 60% of the umbels sampled were from plants grown in the greenhouse at CSUDH. To make sure that the original location of the plants was not driving the differences we observed in the nectar microbial communities we subset the data to only include samples collected from the plants grown at CSUDH and analyzed the microbial community compositions, once again aggregated to the class taxonomic level, visualizing them with an NMDS. We found that the NMDS showed tighter clustering of the SCBG samples and wider variance among the UCR samples similar to what we had seen when all the bacterial samples were compared (PERMANOVA F_2,70_ = 13.723, *P* < 0.001, R^2^ = 0.282; Fig 5A), with a slight difference in the clustering of the CSUF samples that more closely mirrored the SCBG samples. Similarly, our analysis of the distance to the centroid of the sample points with the Wilcoxon signed- rank test found significantly greater variance in the bacterial composition of the UCR samples with much less variance and distance to the centroid in the community composition of the samples from SCBG (*P <* 0.001; Fig 5C). The slight difference in dissimilarity between the analysis of the samples from plants grown at the CSUDH greenhouse and that of all the samples pooled together may have been due to some variation in the samples from the plants native to the gardens (Fig 5B and 5D). Our NMDS ordination denoting site and plant type also supports this (S1 Fig).

## Conclusions

Contrary to past studies that have found butterflies to be the most common visitors to *A*. *curassavica* [44–46], the main visitor to our plants was the non-native honey bee, *Apis mellifera*. Similar to other milkweed species [68], even within a single day we observed a broad taxonomic diversity of floral visitors, including both insects and birds. Many of these have been documented visitors to *A*. *curassavica* in other places, such as ants in the American tropics [44], different species of bees in India [46], and several species of butterflies in Costa Rica [45]. Unique to our observations was the hummingbird, the different types of beetles, the moths, and the different wasps, particularly the tarantula hawk wasp. These different floral visitors, each with their own assortment of microbial communities, potentially introduced new microbes into the nectar, influencing the variety that we observed in the composition of these microbial communities [69].

We observed higher species richness of floral visitors at SCBG (11 different species) than at CSUF (six different species) or UCR (eight different species). There was a significant difference in the ratio of floral visitors to the number of flowers per umbel between the SCBG and CSUF samples which may have been the result of the greater overall visitations at CSUF suggesting the interactions of more floral visitors with each nectar sample at CSUF than at SCBG. This may have played a part in the greater variance in the composition of the communities at CSUF than those at SCBG (Fig 4A and 4C) [34,69]. The lower temperatures during the observation at SCBG along with the higher proportion of cloud cover may have made it a less favorable day to forage for nectar [70,71], whereas the sunny, warm environments at CSUF and UCR may have been more suitable for monarch [72] and honey bee [73] foraging. Due to the high visitation rates, we could not match individual floral visitors directly to the microbial diversity. Tantalizingly we found that there were multiple unique OTUs that were found in umbels that were visited only by honey bees. The most common was *Acinetorbactor* followed by *Caminicella*. Further studies on the individual contributions of the different floral visitors to the microbial community are ongoing.

We found that the microorganisms that exhibited increases or decreases in relative abundance between the three sites were those most commonly found in other nectar microbiome studies [34,74–79]. *Acinetobacter* has been identified as a nectar specialist efficient at producing acid from the metabolism of both glucose and sucrose [80], two sugars commonly found in *A*. *curassavica* nectar [47]. *Neokomagataea*, formerly known as *Gluconobacter*, is also an acid producing nectar-dwelling bacteria [81,82]. Both of these bacteria have been shown to lower the pH of nectar [80,82], and exert priority effects on late arriving yeast nectar specialists, such as *Metschnikowia reukaufii*, [67,83] but their effect on *Aureobasidium*, a yeast generalist very commonly found in nectar [34,77–79], that made up a large proportion of the fungal communities in the nectar samples in this study as well, is yet to be determined. The low yield of yeast across our samples may be a sampling artefact due to the short time frame of sampling [30], as milkweed nectar is known to house multiple species of yeasts [84]. Although none of the botanic gardens we used as sites routinely spray pesticides or insecticides, drift from nearby urban or agricultural areas may have impacted the yeasts but not the bacteria [85,86].

Studies delving into how the overall regional nectar microbial species pool is changing with urbanization and how these changes are affecting the preferences of pollinators for nectar [33,37,38] could increase our understanding of the mechanism behind the observations we have presented in this study. In addition, studies into the differences between the bacterial and fungal communities found in non-native flowering species versus native species, and the community interactions occurring in nectar, such as priority affects [83] or competitive exclusion [34], could help us understand how nectar composition is changing and how this may give an advantage to pollinators who are more flexible in their preferred nectar sources [15,87]. Greater understanding of the role that urban gardens play for connectivity between floral visitors [8,88] and nectar microbes could also help with management and planning of urban gardens, increasing resources for pollinators and helping maintain the diversity of floral visitors [33,38] and the fitness of plants [37,40,67,89].

## Supporting information

S1 Table Second PCR index sequences. The index sequences were adding in the second PCR for multiplexing the samples on the Illumina MiSeq.
(XLSX)

S2 Table Floral visitation to umbels at each site. This table identifies the floral visitors recorded at each of the *Asclepias curassavica* umbels.
(XLSX)

S1 Fig NMDS ordination of bacterial diversity denoting site and plant type. **N**All data was aggregated to the class taxonomic level for this analysis and each point represents the bacterial community in one nectar sample. (A) The NMDS ordination of the bacterial communities and (B) the NMDS ordination of the fungal communities have ellipses that show the 95% confidence interval. (C) The distance to centroid of each bacterial community and (D) the distance to centroid of each fungal community were calculated in multivariate space using betadisper. “***” *P <* 0.001, “**” *P <* 0.01, “*” *P <* 0.05 based on Wilcoxon signed-ranks test for pairwise comparisons.
(TIFF)
