# Supplementary figures and images for: Characterizing the nectar microbiome of the non-native tropical milkweed, Asclepias curassavica, in an urban environment
Source: PLoS One. 2020 Sep 2;15(9):e0237561. doi: 10.1371/journal.pone.0237561 (PMC7467256; doi:10.1371/journal.pone.0237561)

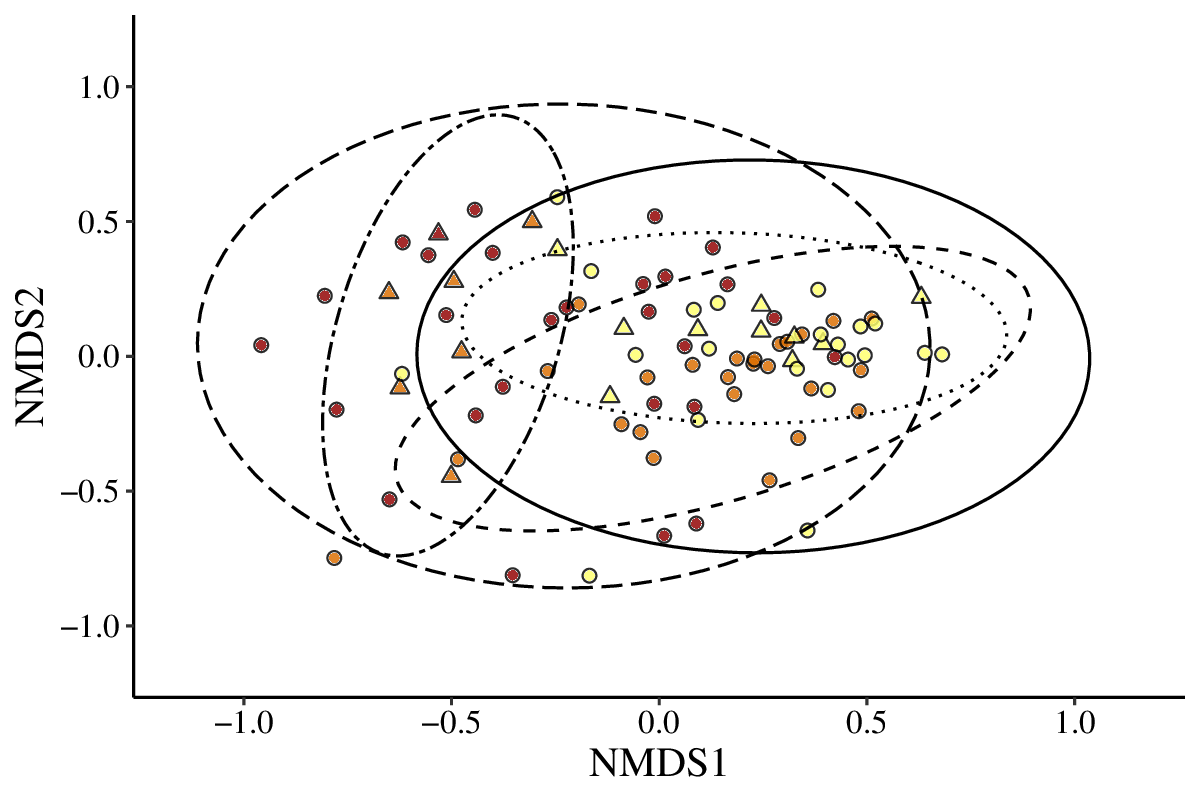

Supplement: S1 Fig — NAll data was aggregated to the class taxonomic level for this analysis and each point represents the bacterial community in one nectar sample. (A) The NMDS ordination of the bacterial communities and (B) the NMDS ordination of the fungal communities have ellipses that show the 95% confidence interval. (C) The distance to centroid of each bacterial community and (D) the distance to centroid of each fungal community were calculated in multivariate space using betadisper. “***” P < 0.001, “**” P < 0.01, “*” P < 0.05 based on Wilcoxon signed-ranks test for pairwise comparisons. (TIFF) [file pone.0237561.s003.tiff]
